# Bounce back from adversity: a narrative review and perspective on the formation and consequences of athlete resilience

**DOI:** 10.3389/fpsyg.2025.1599145

**Published:** 2025-06-16

**Authors:** Zhengyang Mei, Chenyi Cai, Tingfeng Wang, Chifong Lam, Ranran He, Shi Luo

**Affiliations:** ^1^School of Physical Education, Southwest University, Chongqing, China; ^2^School of Physical Education, Shanghai University of Sport, Shanghai, China; ^3^Key Laboratory of Cognition and Personality, Faculty of Psychology, Ministry of Education, Southwest University, Chongqing, China; ^4^Department of Physical Education, Tianjin University, Tianjin, China

**Keywords:** athletes, resilience, formation, consequences, synergistic interactions, positive psychology

## Abstract

In the field of sports, athletes are often exposed to sports adversity or stressful situations. Athlete resilience, as a key psychological factor, is directly associated with athletes’ physical and mental health and sports performance. Despite the growing attention to athlete resilience research, the field still lacks a unified conceptual and theoretical framework to explain the formation and consequences of athlete resilience. These limitations hinder the effective translation of research findings into intervention practices. Reviewing the previous research, this study aimed to provide a narrative review of the definition, structural dimensions, and measurement methods of athlete resilience, and elucidate and analyze its formation, consequences, and synergistic interaction with sports organizational resilience on the basis of theoretical models and relevant theories. Athlete resilience refers to the capacity of athletes to evaluate and regulate their thoughts, emotions, and behaviors in response to sports adversity, thereby enhancing their potential, emotional well-being, and overall health. As a complex multifactorial structure, athlete resilience primarily covers five structural dimensions: sports motivation, self-efficacy, coping strategy, optimism, and hope. At present, the formation of athlete resilience has primarily been studied through frameworks such as the dual-pathway model, meta-model, and psychological immunity-psychological elasticity model, along with their relevant theories. Furthermore, the consequences of athlete resilience are closely associated with various psychological states and behavioral patterns athletes experience during training and competition, the most common of which include perceived stress, competition anxiety, and athlete burnout, and its mechanism can be explained by the meta-model of stress, emotions and performance. Finally, the synergistic development of athlete resilience and sports organizational resilience is crucial, as it effectively enhances the overall ability of athletes and their organizations to cope with and overcome adversity and stress. While existing research has made notable contributions to the understanding of athlete resilience, the field still lacks a more comprehensive and systematic theoretical framework to guide related research. The conceptual foundations, formation and consequences of athlete resilience, along with its synergistic interaction with sports organizational resilience, require further validation and support. This is particularly crucial for enhancing athletes’ overall well-being and their sports performance.

## 1 Introduction

Resilience refers to a key psychological factor that enables individuals to cope with and recover effectively in the face of setbacks and adversity and to maintain the normal operation of physical and mental functions (Antonovsky, 1987; Mei et al., 2024a). Resilience serves as the ability to return to an adaptive and healthy behavioral pattern following adversity (IJntema et al., 2021). Adversity refers to unfavorable or stressful situations encountered by individuals, whose severity is shaped by both the magnitude and duration of exposure (Biron et al., 2006; Bryan et al., 2019). Research indicates that athletes undergo significant changes in their physical and mental states due to the demands of long-term training and competition and face biological, psychological, and social stressors, including training load, injuries, social expectations, fear of failure, coach-athlete relationships, cyber-bullying, and family conflict (Mei et al., 2024b; Sarkar and Fletcher, 2014; Sarkar and Hilton, 2020). The cumulative effect of these stressors considerably increases the risk of developing psychological problem in athletes. According to the latest report of the International Olympic Committee (IOC), between 5 and 35% of athletes worldwide have experienced some form of psychological problem, which refers to localized abnormalities in normal psychological functioning, with common conditions including depression and anxiety; thus, there is an urgent need to provide them with psychological support (Gouttebarge et al., 2021). Positive psychology advocates a focus on the healthy personality and positive qualities of athletes and is committed to using positive psychological resources to promote physical and mental health and quality of life. In this context, athlete resilience (AR), as a psychological factor of positive adaptation, active coping and optimism, can effectively help athletes successfully cope with and overcome adversity and stress, which is crucial to their physical and mental health and career development. Evidence suggests that the development of AR depends on stressors, cognitive evaluation, meta-cognition, and personality and involves five main protective factors (positive personality, motivation, confidence, focus, and social support) that collectively determine the degree to which stressors have a potentially negative impact on athletes (Fletcher and Sarkar, 2012; Sarkar and Hilton, 2020). Therefore, it is imperative to implement effective prevention and intervention measures to improve athletes’ ability to cope with stressors, providing support for theoretical and empirical research on psychological problems in athletes. These findings indicate that AR not only is an important guarantee for the physical and mental health development of athletes but also has significant value in establishing an optimal competitive state and achieving excellent sports performance.

Given the growing academic attention to AR and the complexity of research in this field, this study aimed to synthesize the existing research on the formation and consequences of AR. It seeks to expand insights into its role in athletes’ stress-coping processes and provide theoretical and practical support for the development of resilience intervention strategies, thereby helping athletes improve their physical and mental well-being and quality of life. Specifically, this study aimed to address the following key research questions: (a) How have the definition, structural dimensions, and measurement methods of AR evolved? (b) How does AR develop in individual athletes? (c) What are the consequences of AR at the individual level? and (d) Can AR exert influence at the organizational level?

## 2 Methods

Relevant literature on AR was searched using PubMed, Web of Science, and Google Scholar. The search covered the period from the inception of these databases up until July 2024. Keyword combinations used in the search strategies consisted of (“Resilience” OR “Resilien*”) AND (“Athlet*” OR “Elite*” OR “Sport*”). The detailed search strategy is outlined in Table 1, as per the PubMed database.

**Table 1:** PubMed search strategy.

#1	Resilience [MeSH Terms]
#2	Resilience [Title/Abstract] OR Resilien* [Title/Abstract]
#3	#1 AND #2
#4	Athlet* [Title/Abstract] OR Elite* [Title/Abstract] OR Sport* [Title/Abstract]
#5	#3 AND #4

Studies were included in this narrative review if they met the following criteria: (a) published in English and subjected to peer review; (b) focusing on AR at either the individual or organizational level; and (c) providing qualitative or quantitative analysis and discussion of one or more facets of AR, including definitions, structural dimensions, measurement methods, formation, consequences, and synergistic interaction with sports organizational resilience. Studies were excluded if they (a) did not specifically address resilience; (b) focused solely on general populations without a sport-specific context; and (c) did not discuss AR in terms of its definitions, structural dimensions, measurement methods, formation, consequences, and synergistic interaction with sports organizational resilience. We included high-quality studies that are most relevant and insightful to the narrative focus of this study. This study was informed by our narrative review and experience in the field of AR.

## 3 Results

**Figure 1:**
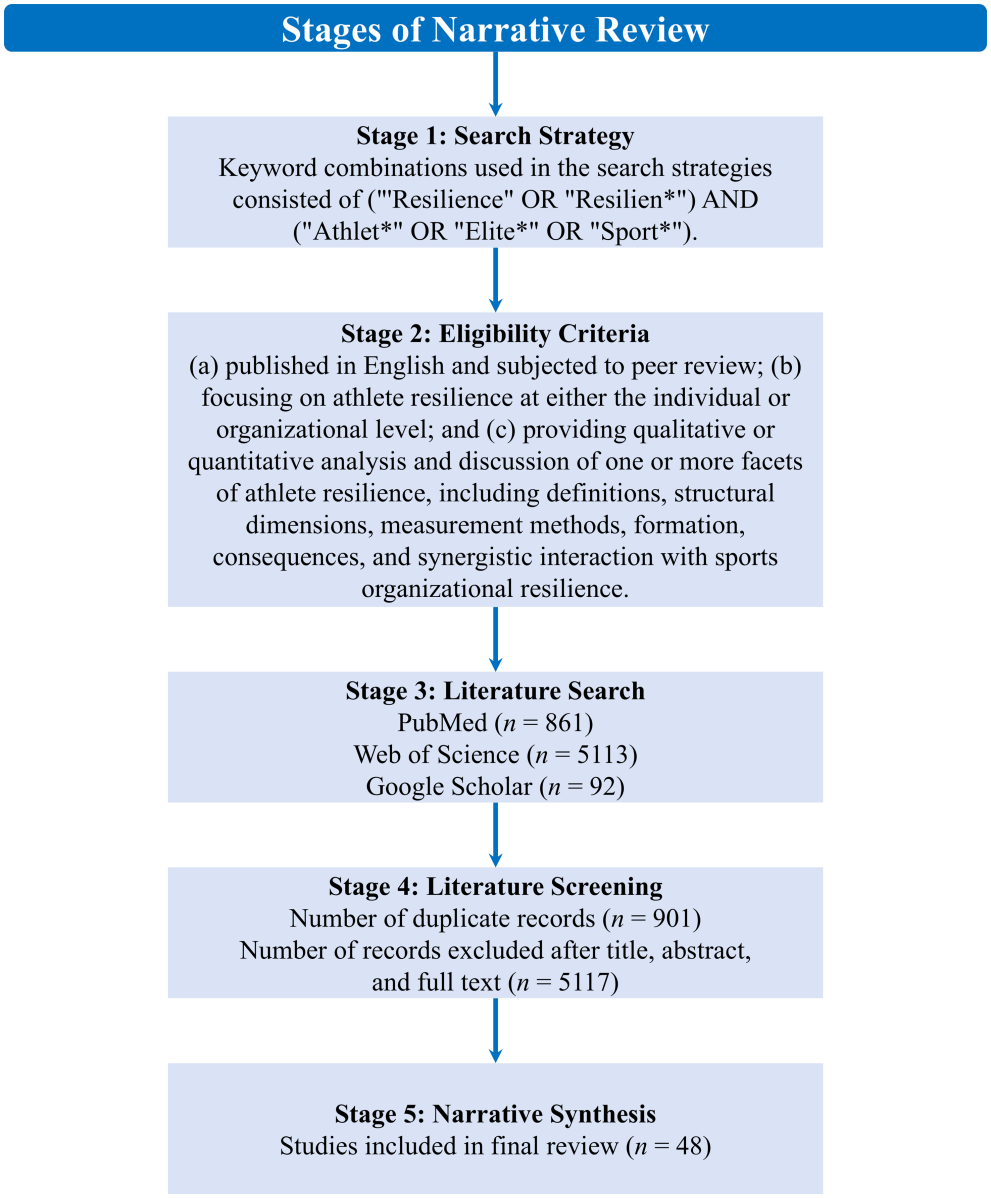
Flow diagram of search results.

A total of 6,066 records were initially identified through literature search, with 861 records sourced from PubMed, 5,113 from Web of Science, and 92 from Google Scholar. After eliminating 901 duplicates, 5,117 records deemed irrelevant to the narrative focus of this study were excluded based on titles, abstracts, and full texts. The final review included 48 studies. The corresponding flow diagram is presented in Figure 1.

### 3.1 Conceptual foundations of athlete resilience

In previous research, differences in the theorization and conceptualization of resilience have led to confusion with other concepts such as “mental toughness,” “hardiness” and “grit” (Andersen, 2012; Galli and Gonzalez, 2015). However, these concepts only connote a “hardy constitution,” absent of any reflective meta-cognitive appraisals, rather than an adaptive response to stress, which is a fundamental way to differentiate them from resilience (Fleming and Ledogar, 2008; Masten et al., 1994). AR was the first generalized by Galli and Vealey (2008), who defined it as the consequence of agitation states caused by athletes’ exposure to stressors managed by sociocultural influences and personal resources. Nevertheless, this definition only regards sports adversity as a practical problem to overcome rather than a challenge and does not consider the subjective assessment of the athlete’s own condition or the dynamic interaction between physical and mental resources and sports adversity, which implies that AR exists only as a passive adaptive process. Subsequently, Fletcher and Sarkar (2012) interviewed 12 Olympic champions about their experiences of adversity based on the grounded theory and defined AR as mental processes and behavior in promoting personal assets and protecting athletes from the potential negative impact of stressors. Recently, there has been a general consensus in the fields of sports and psychology that AR should be recognized as a dynamic process of adaptation to sports adversity (Bryan et al., 2019; Den Hartigh et al., 2022; Fletcher and Sarkar, 2013; Gupta and McCarthy, 2022; Hill et al., 2018a, 2018b; Kalisch et al., 2017; Kegelaers and Sarkar, 2021; Luthar et al., 2000; Métais et al., 2022; Windle, 2011), emphasizing its temporal components and the developmental trajectories that change with sports adversity (Hill et al., 2018b). On this basis, Gupta and McCarthy (2022) defined AR as the capacity of athletes to evaluate and regulate their thoughts, emotions, and behaviors in response to sports adversity, thereby enhancing their potential, emotional well-being, and overall health.

At present, few studies on AR explore its intrinsic structural dimensions and how it is measured. In practice, several self-report scales are commonly employed to assess AR, including the Connor-Davidson Resilience Scale-25 (CD-RISC-25), the Connor-Davidson Resilience Scale-10 (CD-RISC-10), the Resilience Scale (RS), the Brief Resilience Scale (BRS), the Resilience Scale for University Athletes (RSUA), and the Resilience Scale for Adults (RSA; Block and Kremen, 1996; Campbell-Sills and Stein, 2007; Connor and Davidson, 2003; Friborg et al., 2003; Martin and Marsh, 2006; Smith et al., 2008; Tedeschi and Calhoun, 1996; Ueno et al., 2014; Walgnild and Young, 1993). The CD-RISC-25 and the CD-RISC-10 are widely applied in the context of sports. Specifically, the CD-RISC-25 developed by Connor and Davidson (2003) was the first to explore the relevant structural dimensions of AR, consisting of a five-factor structure: personal competence, stress reaction, positive adaptation, perceived control, and spiritual influence. Nevertheless, the validity of the structural dimensions of the CD-RISC-25 has not been supported by empirical research, with some of the dimensions showing low factor loadings (Gonzalez et al., 2016; Gucciardi et al., 2011). In contrast, the CD-RISC-10 provides better model fit and measurement convenience, but it still has major limitations; that is, the scale considers AR as a single-factor structure, and does not cover items related to experiences of adversity and positive adaptations in athletes, which means that the CD-RISC-10 is unable to assess AR effectively (Bicalho et al., 2022; Gonzalez et al., 2016). The measurement of AR involves a dynamic process of interaction between the individual and the environment, which requires an ongoing assessment of changes in athletes’ physical and mental resources and sports adversity (Reppold et al., 2012; Sarkar and Fletcher, 2013; Secades et al., 2016).

Against this background, through a systematic analysis and review of previous literature (Fletcher and Sarkar, 2012; Galli and Vealey, 2008; Gernigon et al., 2010; Lundqvist and Kenttä, 2010; Sarkar and Fletcher, 2014; Sève et al., 2007), Den Hartigh et al. (2022) summarized the structural dimensions of AR from a multidisciplinary, dynamic, and personalized perspective, primarily covering sports motivation, self-efficacy, coping strategy, optimism, and hope. Sports motivation refers to the internal motivation of athletes to engage in or maintain sports, serving as a primary determinant of sports-related behaviors (Pelletier et al., 1995). Self-efficacy refers to athletes’ judgments of their ability to organize and execute the courses of action required to attain a desired outcome, with the potential to shape efforts, affective experiences, and enjoyment of physical activities (Bandura, 1977; Samson and Solmon, 2011). Coping strategy refers to the changing cognitive and behavioral efforts that athletes implement to manage internal and external demands when these demands exceed their available physical and mental resources (Lazarus and Folkman, 1984). Optimism refers to the positive psychological characteristics of athletes, embodying expectations and confidence in future career development, and is associated with positive emotions, mental toughness, sports achievements, and physical and mental health (Peterson, 2000). Hope refers to athletes’ cognitive thought processes aimed at achieving goals, involving setting meaningful and clear goals, along with developing the motivations and strategies necessary for their accomplishments (Snyder et al., 1991). Although the above structural dimensions have been validated in several empirical studies, the scale covering these structural dimensions requires further development and validation to test the scientificity and validity of the five-factor structure of AR.

### 3.2 Formation of athlete resilience

**Figure 2:**
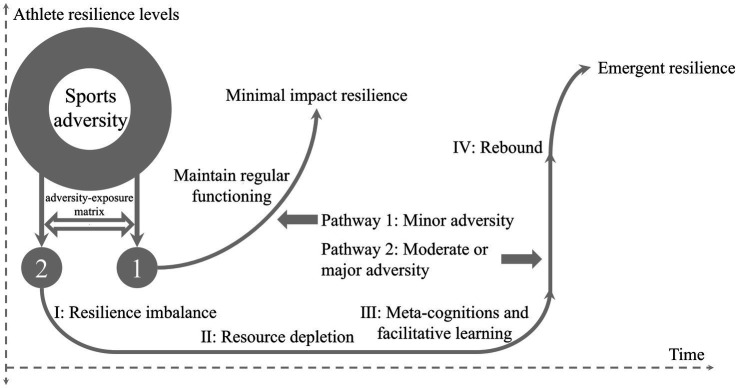
Dual-pathway model of athlete resilience.

**Figure 3:**
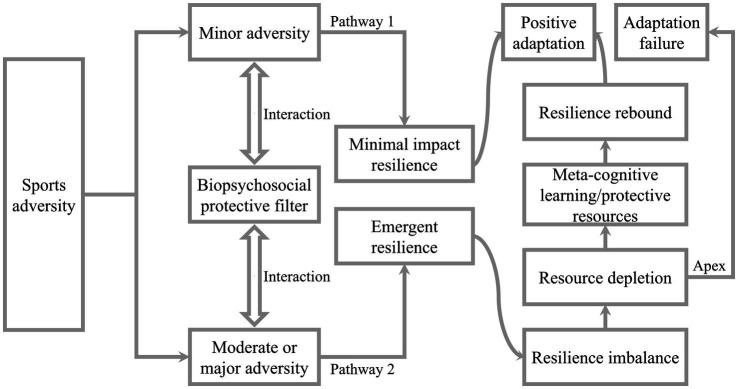
Meta-model of athlete resilience.

**Figure 4:**
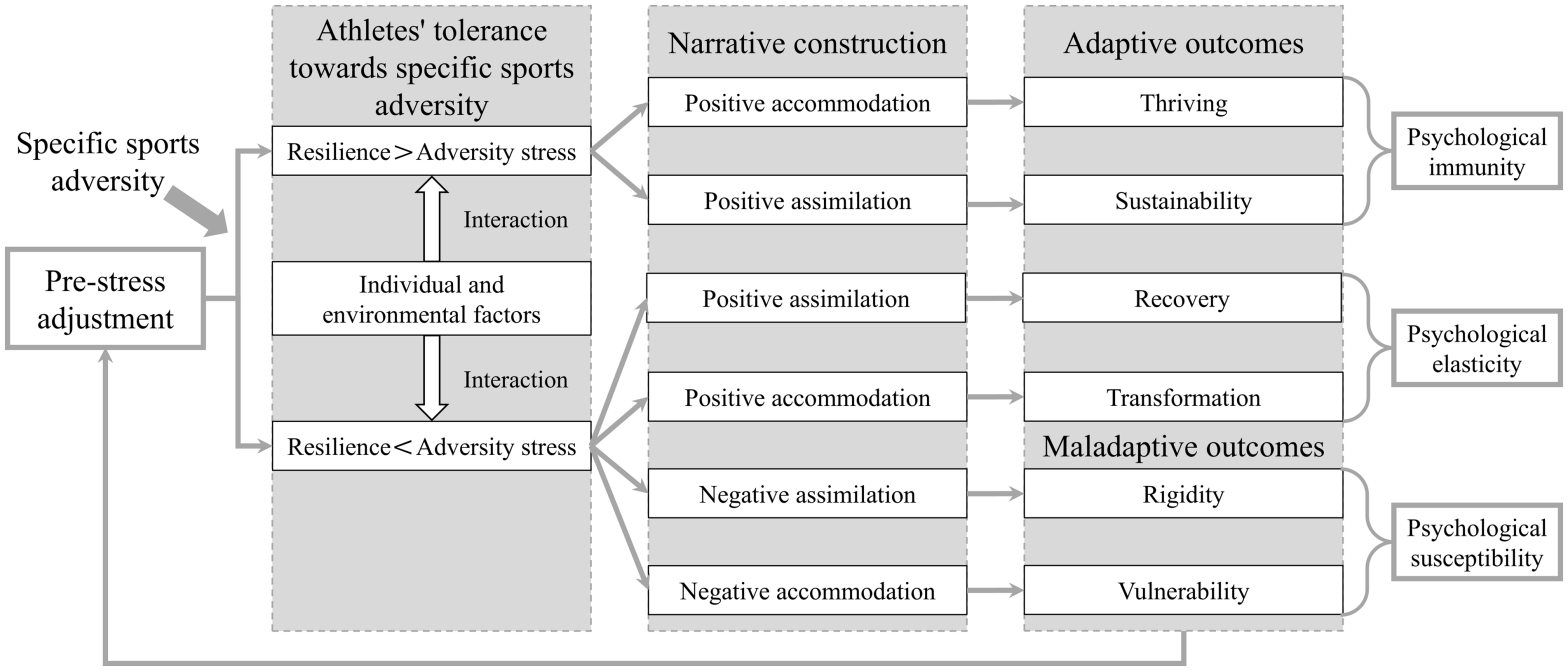
PI-PE model of athlete resilience.

The formation of AR has primarily been studied through frameworks such as the dual-pathway model, meta-model, and psychological immunity-psychological elasticity model, along with their relevant theories. The relevant theoretical models of AR are presented in Figures 2–4.

#### 3.2.1 Dual-pathway model of athlete resilience

Sports adversity (or stressful situations) arises from a combination of biological, psychological, and social stressors (as outlined in Table 2), as well as a necessary condition for the formation of AR (Britt et al., 2016; Luthans et al., 2006). Based on the adversity-exposure matrix, there may be two potential pathways for the formation of AR, namely, minimal impact resilience and emergent resilience (Bonanno and Diminich, 2013). The degree of exposure to adversity depends on the magnitude and duration of the specific adversity, which in different combinations can constitute minor, moderate or major adversity. According to the conservation of resources theory, when encountering minor adversity (a bad training atmosphere, mild injury, etc.), athletes are able to maintain their regular physical and mental functioning by gathering psychological resources (Hobfoll, 2002). Therefore, there is no imbalance in AR due to the presence of adversity exposure, a pathway known as minimal impact resilience. However, when the degree of exposure to adversity reaches a moderate or major level (performance failure, major injury, etc.), athletes’ psychological resources will undergo a process of imbalance, depletion, disruption, and reorganization, which may stimulate a higher AR levels, a pathway known as emergent resilience (Bryan et al., 2019). The establishment of this pathway is primarily based on the broaden-and-build theory, the core idea of which is that the positive adaptive tendencies adopted by athletes during sports adversity can help them build and develop psychological resources and increase AR levels (Fredrickson, 2001). According to the meta-theory of resilience, this enhancement process covers four stages: resilience imbalance, resource depletion, meta-cognitions and facilitative learning, and rebound (Richardson, 2002), each of which reflects a different pattern of changes in athletes’ physical and mental states during adversity exposure (Bryan et al., 2019).

**Table 2:** Sources and specific manifestations of sports adversity.

Sources of sports adversity	Specific manifestations
Biological stressors	Training load, injury and illness, sleep problems, dietary and nutritional problems, etc.
Psychological stressors	Competition pressure, fear of failure, performance failure, self-expectation, self-doubt, work-life balance, etc.
Social stressors	Coach-athlete relationship, tram emotional atmosphere, social expectation, cyber violence, audience effect, family conflict, financial pressure, sports selection, logistical support, career transition, organizational culture, organizational change, etc.

The impact of adversity exposure on AR has been confirmed by previous research. For instance, Brown et al. (2015) reported that the appropriate type and timing of adversity may stimulate higher levels of goal-orientation and self-efficacy in athletes, thereby buffering and counteracting the adverse impact caused by sports adversity, while maintaining regular physical and mental functioning (Pathway 1). Similarly, Sarkar et al. (2015) reported that some maladaptive psychological states (anger, anxiety, etc.) experienced by athletes during adversity do not necessarily lead to long-term negative behavioral responses. Instead, these experiences help athletes stimulate higher AR levels and sports performance after calm and reflective processing (Pathway 2). Notably, the dual-path model of AR regards AR as a dynamic process of ongoing adaptation to varying degrees of sports adversity, influenced by athletes’ psychological and situational resources (Bonanno and Diminich, 2013). Furthermore, this model places particular emphasis on the relationship between sports adversity and different situational demands while underscoring the necessity of sports adversity for the formation of AR (Bryan et al., 2019). However, the dual-pathway model of AR lacks clarity in defining criteria for the magnitude and duration of various types of sports adversity, and it fails to account for the personal differences in athletes during sports adversity. This implies that the model is relatively limited in predicting the response trajectories of AR. It is necessary to further clarify and refine the operational mechanisms, as well as the influence relationships between various internal factors (personal traits, cognitive appraisal, coping ability, etc.) and external factors (organizational environment, team resources, coaching support, etc.) on the development process of AR. Analyzing the differential effects of these factors on AR will help construct a more comprehensive and accurate theoretical framework for explanation. In summary, on the basis of the dual-pathway model of AR, the formation of AR primarily stems from sports adversity, with its development influenced by athletes’ psychological and situational resources.

#### 3.2.2 Meta-model of athlete resilience

AR can be conceptualized as an oscillatory process of athletes in response to sports adversity, which is not an isolated linear process with a discrete start-middle-end (Gupta and McCarthy, 2022), but rather as a dynamic process unfolding over time, thereby giving rise to the concept of “meta” (Bonanno, 2004, 2012; Bonanno and Diminich, 2013; Bryan et al., 2018; Galli and Gonzalez, 2015; Galli and Pagano, 2018; Galli and Vealey, 2008). In addition, the grounded theory of AR and the broaden-and-build theory suggest that the potential adverse impact of stressors on athletes can be buffered by specific physical and mental resources (or protective resources), which serve as “filters” and “buffers” (Fletcher and Sarkar, 2012; Halbesleben et al., 2014). Based on the above, the meta-model of AR constructs the biopsychosocial protective filter (BPF), which encompasses protective resources (positive personality, social support, etc.) that athletes possess in response to sports adversity. These resources can develop over time and through repeated experiences of adversity (Gupta and McCarthy, 2022). The model emphasizes that the response trajectories of AR are determined by the strength of available resources within the BPF and the degree of exposure to adversity (Gupta and McCarthy, 2022). The potential negative impact of sports adversity is counteracted by the BPF in athletes, and as the strength of the BPF increases, athletes may experience reduced harm from sports adversity (Gupta and McCarthy, 2022). This process draws parallel from biological immune systems, where a robust immune system can protect athletes from the impact of illnesses with no adverse disruptions or in a defense process that disrupts internal biological homeostasis, thereby promoting physcial and mental well-being (Kotas and Medzhitov, 2015; Miller and Maner, 2011).

Research has confirmed that when athletes encounter sports adversity, protective resources such as social support from various levels (coaching support, parental support, peer support, etc.) can act as “buffers,” thus reducing, to some extent, the adverse impact of adversity on athletes’ mental health and enhancing their ability to cope with adversity and stress (Lu et al., 2016; Mira et al., 2023). Similarly, a qualitative study exploring athletes’ experiences of adversity confirmed that protective psychological resources—such as a positive attitude, determination, commitment, and perseverance—play a critical role in helping athletes cope with challenges related to poor performance or serious injury and facilitate physical and mental recovery (Galli and Vealey, 2008). In addition, the meta-model of AR posits that the activation of the pathway of minimal impact resilience occurs when the strength of the BPF is adequate to buffer or filter the potential adverse impact resulting from sports adversity, facilitating athletes’ positive adaptations to adversity (Gupta and McCarthy, 2022). However, when the strength of the BPF is inadequate to fully offset these potential adverse impacts, the response trajectories of AR may manifest in two scenarios: positive adaptation and adaptation failure (Gupta and McCarthy, 2022). The strength of the BPF determines the “AR apex,” and if this apex (the maximum value of emergent resilience) can be dynamically stabilized with sports adversity, athletes will re-establish dynamic equilibrium with their environment by identifying and utilizing existing and new resources (Gupta and McCarthy, 2022). In contrast, if this apex is beached by specific sports adversity (high-intensity and prolonged stressful situations, etc.), the response trajectories of emergent resilience may enter a second phase, triggering sustained resource depletion and a downward negative spiral, ultimately leading to adaptation failure (Fredrickson, 2001). In summary, on the basis of the meta-model of AR, the formation of AR primarily stems from sports adversity, with its development influenced by the BPF.

#### 3.2.3 Psychological immunity-psychological elasticity model of athlete resilience

The psychological immunity-psychological elasticity (PI-PE) model explains the formation of AR on the basis of the interactions between athletes’ behavior, cognition, and emotions in specific times and contexts (IJntema et al., 2019; IJntema et al., 2021). The PI-PE model consists of two mechanisms, three conditions, and two outcomes, and the specific components and their interpretations are presented in Tables 3, 4 (IJntema et al., 2021). Studies have confirmed that sports adversity can be viewed as a precondition or triggering factor for the response trajectories of AR, indicating that AR cannot develop without sports adversity (Bonanno et al., 2015; Fletcher and Sarkar, 2013; Windle, 2011). The PI-PE model assumes that AR is not capable of being generated in any sports adversity but exists within specific sports adversity (IJntema et al., 2021). Furthermore, since the response trajectories of AR depend on athletes’ tolerance toward specific sports adversity, athletes may differ in their tolerance toward different types of sports adversity, and this process is also influenced by athletes’ pre-stress adjustment (the extent to which athletes are psychologically adapted to specific sports adversity prior to exposure to it), individual and environmental factors (IJntema et al., 2021). Athletes can develop tolerance toward specific sports adversity by successfully coping with them, enabling better adaptation and coping in the future. This process is known as the steeling effect of adversity (Rutter, 1985, 2012). For instance, Brown et al. (2015) confirmed that after experiencing a specific sports adversity, athletes can increase their tolerance to this adversity by analyzing and applying the knowledge and experience gained from it, thereby reducing maladaptive psychological and behavioral responses. The opposite of the steeling effect of adversity is the sensitizing effect of adversity, where athletes fail to successfully cope with specific sports adversity, resulting in maladaptive outcomes and making athletes more susceptible to similar future sports adversity (Rutter, 1985, 2012).

**Table 3:** Specific components of PI-PE model.

Mechanisms	Conditions	Outcomes	
		Adaptive	Maladaptive
Athletes’ tolerance toward specific sports adversity	Pre-stress adjustment (Reference point for adaptation to sports adversity)	Thriving	Rigidity
	Sports adversity (Key condition for triggering AR)	Sustainability	
Narrative construction	Individual and environmental factors (Influential factors regarding sports adversity, mechanisms, and outcomes)	Recovery	Vulnerability
		Transformation	

AR, Athlete resilience.

**Table 4:** Interpretations of all concepts and their functions in PI-PE model.

Concepts	Interpretations	Functions
Pre-stress adjustment	The extent to which athletes are psychologically adapted to specific sports adversity prior to exposure to it	A set-point for interpreting the outcome of the response trajectories of AR
Specific sports adversity	A specific demanding or difficult situation faced by athletes	Stimulus for triggering the response trajectories of AR
Tolerance toward specific sports adversity	The extent to which athletes refrain from responding defensively to specific sports adversity	The immediate response after exposure to specific sports adversity and the first phase of the response trajectories of AR
Narrative construction	The extent to which athletes make sense of and come to terms with stressful experiences	The second phase of the response trajectories of AR
Individual and environmental factors	Internal and external factors that influence athlete’s pre-stress adjustment, tolerance, narrative construction, and adaptation to specific sports adversity	To confirm that the response trajectories of AR are dynamic processes interacting with both the individual and the environment
Positive accommodation	Creating a new narrative which is constructive for the self or the environment to incorporate a stressful experience	A type of narrative construction
Positive assimilation	Incorporating a stressful experience into an existing narrative which is constructive for the self or the environment	A type of narrative construction
Negative accommodation	Creating a new narrative which is unconstructive for the self or the environment to incorporate a stressful experience	A type of narrative construction
Negative assimilation	Incorporating a stressful experience into an existing narrative which is unconstructive for the self or the environment	A type of narrative construction
Thriving	Increased AR compared with pre-stress period	Adaptive outcome
Sustainability	Maintained AR compared with pre-stress period	Adaptive outcome
Recovery	AR rapidly returns to previous level after exposure to sports adversity	Adaptive outcome
Transformation	Changed AR through narrative reconstruction after exposure to sports adversity	Adaptive outcome
Rigidity	Ineffective responses to sports adversity due to restricted AR	Maladaptive outcome
Vulnerability	Decreased AR compared with pre-stress period	Maladaptive outcome
Psychological immunity	Pre-stress adjustment is robust enough to tolerate specific sports adversity	Adaptive pathway
Psychological elasticity	To adapt to specific sports adversity through narrative construction after exposure to sports adversity	Adaptive pathway
Psychological susceptibility	Difficulty in adapting to specific sports adversity through narrative construction after exposure to sports adversity	Maladaptive pathway

AR, Athlete resilience.

On the basis of the organismic valuing theory of growth through adversity, the PI-PE model distinguishes athletes’ tolerance toward specific sports adversity into two processes: accommodation and assimilation (narrative construction; Joseph, 2009; Joseph and Linley, 2005). When the degree of sports adversity faced by athletes is not sufficient to disrupt their core narratives (i.e., AR exceeds adversity pressure), these stressful experiences can be integrated into the existing narratives without changing their core narratives but rather can change only the interpretation or meaning of the event (a specific sports adversity, etc.). This process is known as assimilation (IJntema et al., 2021). However, in situations where sports adversity is sufficient to disrupt the core narratives (i.e., adversity pressure exceeds AR), assimilation becomes difficult. In that case, athletes need to change their existing narratives and construct a new narrative about themselves or their environment by learning from specific sports adversity. This process is known as accommodation (IJntema et al., 2021). Notably, assimilation and adaptation have positive and negative aspects, with the positive aspects being associated with adaptive outcomes for athletes, including thriving, sustainability, recovery, and transformation. In contrast, the negative aspects are associated with maladaptive outcomes, including rigidity and vulnerability (Brandtstädter and Rothermund, 2002; Leipold and Greve, 2009). At present, the PI-PE model remains in the theoretical exploration stage, and there is a lack of corresponding empirical research supporting and validating its core viewpoints and mechanisms. This suggests the need for empirical and applied work based on the PI-PE model to examine its theoretical validity and practical applicability in explaining the formation of AR and its outcomes. In summary, on the basis of the PI-PE model, the formation of AR primarily stems from athletes’ pre-stress adjustment and specific sports adversity, with its development being influenced by individual and environmental factors, which may lead to various psychological states and behavioral patterns in athletes.

### 3.3 Consequences of athlete resilience

The consequences of AR are closely associated with various psychological states and behavioral patterns athletes experience during training and competition, the most common of which include perceived stress, competition anxiety, and athlete burnout. For instance, in terms of perceived stress, a cross-sectional study of Spanish athletes suggested that athletes with lower AR levels tend to have higher levels of perceived stress and tend to adopt negative coping strategies in stressful situations (Secades et al., 2016). This adverse cognitive-behavioral pattern significantly limits sports performance. Conversely, a controlled trial examining the effects of an AR training program on the mental health of college athletes revealed that improvements in AR can help athletes actively adopt adaptive coping strategies in the face of stressful events, and can effectively reduce athletes’ perceived stress and increase their sense of well-being (Sullivan et al., 2023). Similarly, a cross-sectional study of Brazilian athletes indicated that higher AR levels can effectively mitigate athletes’ perceived stress, including general stress, emotional stress, and social stress, and enhance their ability to recover from sports adversity (Codonhato et al., 2018). In terms of competition anxiety, a cross-sectional study involving Chinese table tennis athletes revealed that athletes with higher AR levels exhibit less pre-competition cognitive anxiety and better sports performance than athletes with lower AR levels (Zhang et al., 2024). These findings are consistent with evidence from previous studies that the relationship between stressors and competition anxiety can be moderated by AR (Wu et al., 2022). Specifically, as AR levels increase, the induced effect of stressors on competition anxiety diminishes accordingly, suggesting that AR helps alleviate the negative emotions triggered by stressors. In terms of athlete burnout, a prospective study of young elite athletes indicated that athletes with higher AR levels show fewer symptoms of athlete burnout and depression when exposed to high-pressure environments (Gerber et al., 2018). Similarly, a two-wave longitudinal study of adolescent athletes demonstrated that higher AR levels can predict reductions in burnout levels over a 3-month period, thus potentially preventing athlete burnout to some extent (Madigan and Nicholls, 2017). The above studies confirm that enhancing AR may help buffer or counteract the adverse impact on athletes’ physical and mental states caused by exposure to sports adversity or high-pressure environments, thereby improving sports performance (Lu et al., 2016; Simon et al., 2024).

**Figure 5:**
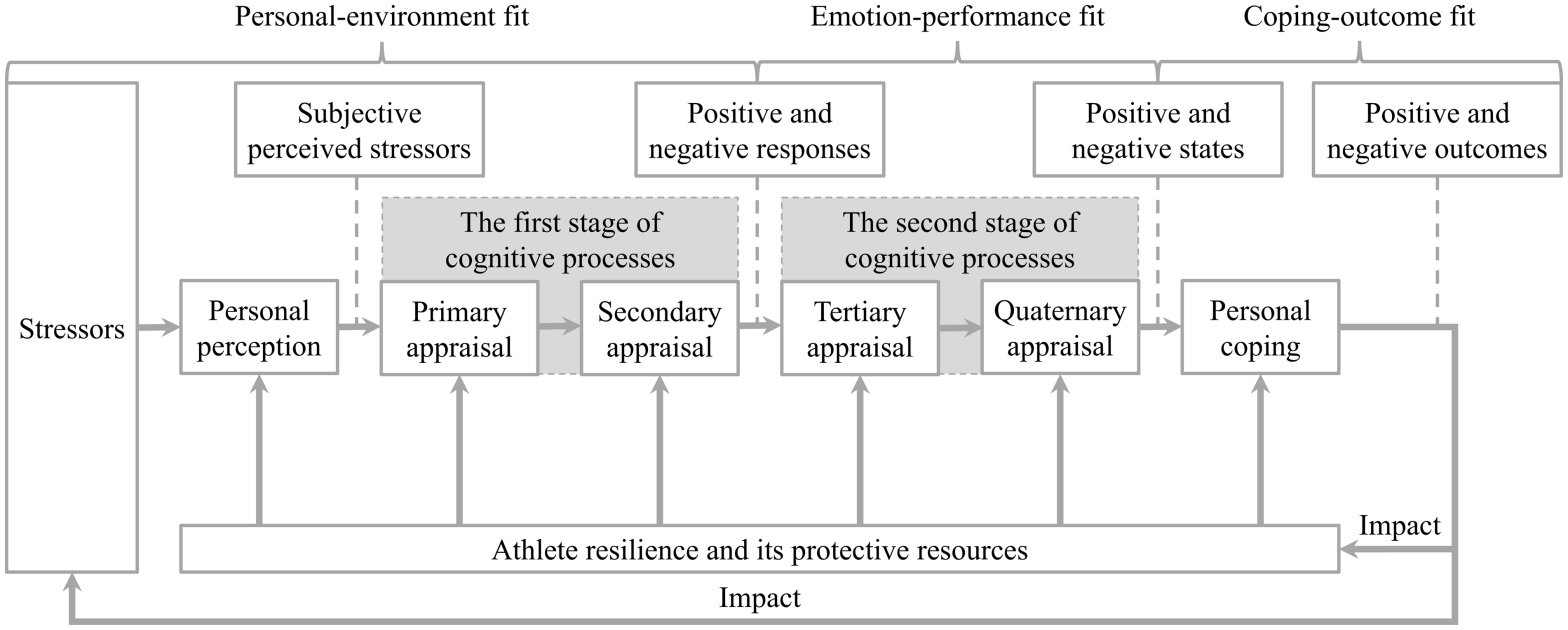
Meta-model of stress, emotions and performance.

The impact mechanism of AR on athletes’ psychological states and behavioral patterns can be explained by the meta-model of stress, emotions and performance (see Figure 5) and its relevant theories. The specific components of the model and their interpretations are presented in Table 5. The meta-model of stress, emotions and performance divides the process regarding the impact of stressors on athletes into three stages: (a) the personal-environment fit stage; (b) the emotion-performance fit stage; and (c) the coping-outcome fit stage, which represent athletes’ perceptions and evaluations of stressors, emotional responses, and coping effectiveness, respectively (Fletcher et al., 2008). The model suggests that stress originates from sports adversity to which athletes are exposed to. Influenced by personal perception, appraisal, and personal coping, this stress triggers corresponding responses, states, and outcomes. For instance, in the initial stage, athletes may experience negative emotions such as anxiety, depression, and fear of failure when they perceive that their personal resources are insufficient to meet situational demands (e.g., exposure to sports adversity). This process is also influenced by individual factors (e.g., AR) and situational factors (e.g., social support), leading to differences in stress responses among athletes when confronted with sports adversity (Fletcher et al., 2008). This implies that AR and its protective resources can mediate negative emotions and poor sports performance caused by stressors to a certain extent. This viewpoint aligns with the grounded theory of AR, which suggests that AR can prompt athletes to develop more positive meta-cognitive appraisals and feedback during sports adversity, and to collect more protective resources to increase their ability to cope with sports adversity (Fletcher and Sarkar, 2012). In summary, through research on the consequences of AR, it can be inferred that when athletes encounter sports adversity, AR and its protective resources can mediate the impact of stressors on athletes’ psychological states and behavioral patterns. However, few studies have explored potential mediators and moderators and their influencing pathways in the process of how AR affects athletes’ psychological states and behavioral patterns. This indicates that relevant influential factors and their mechanisms in this process should be further explored on the basis of the existing models and theories, in a bid to examine the effects of AR on athletes’ physical and mental health and sports performance from a more comprehensive and systematic perspective.

**Table 5:** Specific components and their interpretations of the meta-model of stress, emotions and performance.

Specific components	Interpretations
Personal perception	Athletes’ subjective perception of stressors
Primary appraisal	Evaluating the relevance of situational demands (e.g., exposure to sports adversity) to oneself
Secondary appraisal	Evaluating whether personal resources can meet situational demands
Tertiary appraisal	Evaluating the relationship between emotions reactions and sports performance
Quaternary appraisal	Evaluating specific measures to control and cope with emotional reactions
Personal coping	Adaptive or maladaptive behavior patterns adopted by athletes in response to stressors

### 3.4 Athlete resilience in context: interaction with sports organizational resilience

AR is not limited solely to athletes themselves but can also serve as a key psychological factor at the organizational level. While some sports organizations have been able to cope successfully with the adverse impact of sports adversity, others may suffer serious disruption and damage, which suggests the critical importance of AR in enabling sports organizations to respond effectively to adversity and maintain stable development (Morgan et al., 2017). Sports organizational resilience (SOR) refers to the dynamic ability of sports organizations to successfully cope with adversity and stress. It emerges from multi-level (athletes, coaches, and managers) interacting characteristics, enabling sports organizations to prepare for, adapt to, and learn from adversity (Fasey et al., 2021). Based on various qualitative research methods such as focus group interviews and the Delphi method, the main characteristics of SOR have been explored by different scholars, and the research findings are presented in Table 6. At present, SOR is primarily measured via the Characteristics of Resilience in Sports Teams Inventory (CREST), which consists of two dimensions: resilient characteristics (the ability of sport organizations to cope with and overcome adversity) and vulnerabilities under pressure (the weaknesses exposed when sports organizations fail to cope with and overcome adversity; Decroos et al., 2017). Evidence suggests that the two dimensions of SOR are independently correlated rather than opposite endpoints of the same dimension, implying that sports organizations with higher vulnerabilities under pressure do not necessarily lack resilient characteristics (Kegelaers et al., 2021). Therefore, in the process of empirical or applied research, it is crucial to thoroughly consider the complex relationships between different structural dimensions of SOR and variables such as coping strategies and sports performance to further investigate the potential impact and underlying mechanisms of resilience on athletes and sports organizations and provide guidance for the development of targeted interventions.

**Table 6:** Main characteristics and their interpretations of sports organizational resilience.

Study ID	Main characteristics	Interpretations
Morgan et al. (2013)	Group structure	The conventions that shape group norms and roles, and involves both psychosocial and physical aspects
	Mastery approaches	The shared attitudes and behaviors that promote an emphasis on team improvement
	Social capital	The existence of high quality interactions and caring relationships within groups
	Collective efficacy	The group’s shared beliefs in its ability to perform a task
Fasey et al. (2021)	Structural clarity	The need for sports organization to have a clear and effective structure, particularly regarding communication channels, roles and responsibilities between individuals and teams, and decision making
	Flexible improvement	The ability of sports organizations to engage in learning and innovative adaptation, and the flexibility to apply diverse approaches
	Shared understanding	Sports organizations establish a shared belief and code of conduct to achieve collective goals, including a unified vision and values
	Reciprocal commitment	Coaches and athletes develop a two-way allegiance within which athletes feel valued, supported and safe
	Operational awareness	The capability to identify and assess the range of options available to the organization through understanding the operating environment, available resources, and alternative viewpoints

Research has confirmed that the synergistic development of AR and SOR is crucial, as it effectively enhances the overall ability of athletes and their organizations to cope with and overcome adversity and stress. Specifically, a cross-sectional study based on a multi-level analysis revealed that at the individual level, resilient characteristics have a positive predictive effect on subjective performance perceptions (both athletes and teams), whereas vulnerabilities under pressure negatively predict it. At the team level, resilient characteristics have a positive predictive effect on team sports performance, but vulnerabilities under pressure are not associated with it (López-Gajardo et al., 2023a). Similarly, a longitudinal study of collective sports indicated that resilient characteristics and vulnerabilities under pressure can serve as positive and negative predictors of perceived team performance, respectively, and that both cohesion and collective efficacy can enhance resilience and strengthen the ability of sports organizations to successfully cope with and overcome adversity and stress (López-Gajardo et al., 2023b). The above empirical research suggests that the interactions of individual and team resources in collective sports directly affect sports performance at the individual and team levels, implying that promoting the development of SOR is necessary. An ethnography-based qualitative study revealed psychosocial enablers and strategies for the development of SOR, and the research findings are presented in Table 7 (Morgan et al., 2019). In summary, the synergistic development of resilience at different levels is crucial for athletes and their sport organizations to improve their psychological quality and sports performance. During training and competition, it is essential to further identify and clarify the key driving factors and protective resources associated with different levels of resilience. This will allow for a deeper exploration of the relationship between resilience at various levels and outcomes, such as individuals’ or organizations’ coping abilities and sports performance, as well as potential mediating or moderating mechanisms. Such insights will help in developing targeted strategies to enhance resilience, reducing the negative impact of sports adversity on athletes and their organizations, ultimately improving their overall well-being and sports performance.

**Table 7:** Psychosocial enablers and strategies of sports organizational resilience.

Psychosocial enablers	Strategies
Team culture	①Inspiring, motivating, and challenging team members to achieve performance excellence ②Develop a team-regulatory system based on ownership and responsibility ③Cultivate a team identity and a togetherness based on a selfless culture ④Expose the team to challenging training and unexpected/difficult situations ⑤Promoting enjoyment and keeping a positive outlook during stressors
Enjoyment in sports	
Identification	

## 4 Discussion

This study provides a narrative review of the definition, structural dimensions, and measurement methods of AR, and elucidates and analyzes its formation, consequences, and synergistic interaction with SOR based on theoretical models and relevant theories. AR refers to the capacity of athletes to evaluate and regulate their thoughts, emotions, and behaviors in response to sports adversity, thereby enhancing their potential, emotional well-being, and overall health (Gupta and McCarthy, 2022). As a complex multifactorial structure, AR primarily covers five structural dimensions: sports motivation, self-efficacy, coping strategy, optimism, and hope (Den Hartigh et al., 2022). At present, the formation of AR has primarily been studied through frameworks such as the dual-pathway model, meta-model, and psychological immunity-psychological elasticity model, along with their relevant theories. (e.g., the conservation of resources theory and the broaden-and-build theory; Bryan et al., 2019; Fletcher and Sarkar, 2012; Fredrickson, 2001; Gupta and McCarthy, 2022; Halbesleben et al., 2014; Hobfoll, 2002; IJntema et al., 2021; Joseph, 2009; Joseph and Linley, 2005; Richardson, 2002). Furthermore, the consequences of AR are closely associated with various psychological states and behavioral patterns athletes experience during training and competition, the most common of which include perceived stress, competition anxiety, and athlete burnout, and its impact mechanism can be explained by the meta-model of stress, emotions and performance (Fletcher et al., 2008). Finally, the synergistic development of AR and SOR is crucial, as it effectively enhances the overall ability of athletes and their organizations to cope with and overcome adversity and stress. Overall, while many valuable results have been obtained in research on the formation and consequences of AR, several unresolved issues warrant attention in future studies.

In terms of the formation of AR, although the dual-pathway model, meta-model, PI-PE model, along with their relevant theories, explain to a certain extent the antecedents and influential factors during the development of AR, a more comprehensive and systematic framework and explanation is still lacking. Most of the existing studies analyze the response trajectories of AR based on sports adversity and the meta-theory of resilience, which limits the understanding regarding the formation of AR. For instance, does AR still exist under non-sports adversity? What are the proximal factors of AR? How do these proximal factors relate to the formation of AR? What individual or situational factors influence the developmental process (changes in resources and narrative construction, etc.) of AR? The aforementioned issues highlight the existing gaps and limitations in the current research on AR, and it is urgent to conduct in-depth analysis to comprehensively understand the internal operational mechanisms of AR and to develop more scientific and effective interventions for athletes to successfully cope with and overcome adversity and stress.

In terms of the consequences of AR, current research has focused primarily on aspects such as perceived stress, competition anxiety, and athlete burnout; however, the possible impact of AR on other aspects have not been fully explored. This partial exploration to some extent restricts the development of applied research on AR. During training and competition, athletes may exhibit various psychological states and behavioral patterns due to variations in their AR, including mental fatigue, adversity belief, sports engagement, sports achievement, training and competition satisfaction. These factors significantly influence sports performance and merit further attention and exploration. In addition, some studies concerning the consequences of AR are cross-sectional in design, implying that the longitudinal causal relationships between AR and these outcomes remain unclear. There is a need for further development of longitudinal samples or intervention experiments to investigate and validate the relationships between AR and relevant outcomes. On this basis, the scope of research on the consequences of AR should be expanded, delving deeper into the dynamic causal relationships in this process, which will enrich and improve the theoretical models of AR, and provide a more comprehensive theoretical foundation for practical applications.

In terms of the synergistic interaction, the emergence of SOR means that AR no longer acts as an isolated individual characteristic, but rather as a result of interaction between athletes and sports organizations. The synergistic development of AR and SOR is crucial, as it effectively enhances the overall ability of athletes and their organizations to cope with and overcome adversity and stress. However, current research in this regard is still in the preliminary exploration and validation stage, and the underlying mechanisms of their synergistic development and effects on athletes and organizations require further investigation and clarification. Furthermore, several unique factors may have multiple interactions with AR and SOR, including coach-athlete relationship, coaching style, and tram training atmosphere, which also warrant attention and examination. Overall, the mechanisms of the synergistic interaction between AR and SOR should be further analyzed to examine how this synergy promotes athletes’ mental health and sports performance at different levels. This process of interaction will certainly be influenced by mediating or moderating factors, indicating that more potential impact should be paid attention to in the study of synergy to better facilitate positive interactions between athletes and their sports organizations through such synergies, pooling resources and strengths to address challenges, thereby achieving long-term prosperity and development.

The insights provided in this review on the formation and consequences of AR hold significant implications for for sports practice, particularly in athlete development. Based on established theoretical models and relevant theories of AR, it is recommended that coaches and psychotherapists design and implement targeted intervention strategies to foster AR. Drawing from theories of stress inoculation (Meichenbaum and Deffenbacher, 1988), it has been suggested that athletes can enhance their resilience by exposing themselves to stressful situations during training or competition. This indicates that resilience intervention strategies should be a key direction for research and practice to effectively enhance athletes’ ability to cope with adversity and stress. More importantly, the synergistic interaction between AR and SOR highlights the necessity of a systemic intervention approach. This implies that resilience intervention strategies should be implemented at both the individual and organizational levels. Such an approach not only enhances the effectiveness of psychological support for athletes and their teams but also contributes to the development of a team culture grounded in resilience. Notably, this review adopts a narrative approach, which allows for a flexible and diverse interpretative synthesis of the existing evidence. Although this approach is well-suited for exploring complex concepts like AR, it also has certain limitations. The lack of systematic inclusion and exclusion criteria may introduce selection bias in the literature, and the interpretative synthesis is also prone to subjective judgment and inference. Therefore, future research could benefit from employing systematic reviews or meta-analyses to validate and quantify the trends identified in this study.

## 5 Conclusion

While existing research has made notable contributions to the understanding of AR, the field still lacks a more comprehensive and systematic theoretical framework to guide related research. The conceptual foundations, formation and consequences of AR, along with its synergistic interaction with SOR, require further validation and support. This is particularly crucial for enhancing athletes’ overall well-being and their sports performance. For researchers, this review identifies key gaps in the field of AR, which contribute to a better understanding of AR’s critical role in athletes’ stress-coping processes. For practitioners, the findings of this review provide guidance for the design and implementation of interventions, laying the foundation for the practical application of AR in the sports context. Overall, this review provides important insights by synthesizing existing theoretical and empirical findings, contributing to the development of a systematic AR research framework and promoting the advancement of the field toward a more precise and applied direction.
